# A novel smartphone-based activity recognition modeling method for tracked equipment in forest operations

**DOI:** 10.1371/journal.pone.0266568

**Published:** 2022-04-06

**Authors:** Ryer M. Becker, Robert F. Keefe

**Affiliations:** Department of Forest, Rangeland and Fire Sciences, College of Natural Resources, University of Idaho, Moscow, Idaho, United States of America; Second Institute of Oceanography Ministry of Natural Resources, CHINA

## Abstract

Activity recognition modelling using smartphone Inertial Measurement Units (IMUs) is an underutilized resource defining and assessing work efficiency for a wide range of natural resource management tasks. This study focused on the initial development and validation of a smartphone-based activity recognition system for excavator-based mastication equipment working in Ponderosa pine (*Pinus ponderosa*) plantations in North Idaho, USA. During mastication treatments, sensor data from smartphone gyroscopes, accelerometers, and sound pressure meters (decibel meters) were collected at three sampling frequencies (10, 20, and 50 hertz (Hz)). These data were then separated into 9 time domain features using 4 sliding window widths (1, 5, 7.5 and 10 seconds) and two levels of window overlap (50% and 90%). Random forest machine learning algorithms were trained and evaluated for 40 combinations of model parameters to determine the best combination of parameters. 5 work elements (*masticate*, *clear*, *move*, *travel*, and *delay*) were classified with the performance metrics for individual elements of the best model (50 Hz, 10 second window, 90% window overlap) falling within the following ranges: area under the curve (AUC) (95.0% - 99.9%); sensitivity (74.9% - 95.6%); specificity (90.8% - 99.9%); precision (81.1% - 98.3%); F1-score (81.9% - 96.9%); balanced accuracy (87.4% - 97.7%). Smartphone sensors effectively characterized individual work elements of mechanical fuel treatments. This study is the first example of developing a smartphone-based activity recognition model for ground-based forest equipment. The continued development and dissemination of smartphone-based activity recognition models may assist land managers and operators with ubiquitous, manufacturer-independent systems for continuous and automated time study and production analysis for mechanized forest operations.

## Introduction

Time and motion studies are used widely in forest operations and provide insight for improving efficiency and reducing delays in forest operations through the observation, analysis, and quantification of productive cycles and work elements [1–11]. Traditionally, harvest cycle times and individual elements are estimated using stopwatches, video recorders and handheld data loggers [12]. These studies are often labor intensive and cost prohibitive due to the manual, observational sampling they require. The application of production functions and treatment cost simulation models developed from time and motion studies is often limited in scope due these constraints [13, 14]. The methods by which productive work is defined in forestry and natural resource management are rapidly evolving, where traditional sampling techniques are increasingly replaced by less labor-intensive methods resulting from advances in real-time spatial data collection, activity recognition, remote sensing, and wearable and mobile technologies [15].

In forest operations, global navigation satellite systems (GNSS) have been successfully used in various forestry applications to automate the sampling and production analysis of equipment, monitor the movements of ground workers, and assess equipment and ground-worker interactions for both safety and production purposes in lieu of or in support of traditional time and motion methods [16, 17]. GNSS-supported approaches for production analysis have been developed for timber harvesting equipment including skidders [18–22], forwarders [14], and cable logging carriages [23]. GNSS devices work particularly well for equipment such as forwarders and skidders due to the large distances they cover and the relationship of their movements, speed, and location with their productive activities [14]. In harvester studies, Standard for Forest Data (StandForD) and CAN bus data have been used in combination with GNSS devices to perform production analysis [12, 24–26]. Further, Carter et al. successfully used GNSS to monitor traffic intensity and soil impacts resulting from timber harvesting [27].

One limitation of many basic GNSS devices is the inability to share and process data in real-time independently of additional hardware, limiting their usefulness for production analysis where immediate feedback and processing may be desired. GNSS-RF uses radio frequency to transmit and share data in real-time independent of cellular and satellite infrastructure, enabling data sharing in remote work environments and off-the-grid scenarios. The use of these technologies for improving safety and situational awareness in forest management and logging scenarios has received significant interest [28–31]. Further, real-time communication between multiple GNSS-RF devices has successfully been used to assess independently moving components of a single machine to characterize productive work elements [32, 33]. Classification of component work elements using consumer-grade GNSS-RF is limited by the spatial resolution and accuracy at which data can be recorded [32, 33]. GNSS accuracy degradation due to multipath error from dense forest canopies or topography further limits the extent spatially explicit data can be used for production analysis in forest operations [14, 16, 19, 34]. This may prohibit the exclusive use of GNSS-based devices for data collection and production representation where high-accuracy position, navigation, and timing (PNT) data are required to ensure worker safety and effective work assessment [35], although these studies predated recent advances in RTK system availability for mobile devices. When GNSS error is unacceptably large, additional data inputs may be necessary to increase the robustness of production models and ensure their improved accuracy and utility across varying sites and applications. Sensor-based data, including those derived from triaxial accelerometers and gyroscopes, provide a rich, high-resolution, and highly descriptive data source for characterizing equipment activities. Wearable and mobile devices, including smartphones and smartwatches, are regularly equipped with inertial measurement units (IMUs), GNSS chipsets, and other sensors that provide detailed information regarding the environment in which the device is located [36, 37]. Inertial measurement units (IMU) contain both a triaxial accelerometer and gyroscope and enables the measurement of acceleration and angular velocity along three orthogonal axes and are regularly used in activity recognition modelling [38].

Activity recognition refers to the prediction of an individual’s actions based on sensor-derived data capturing their movements or other biophysical characteristics and was first deployed in the lifecare and wellness industries in the 1980’s [39]. Smartphone and smartwatch ubiquity has led to rapid expansion in their use for human activity recognition modeling techniques characterizing physical activities. Data collected from smart device-based accelerometers, gyroscopes and sound pressure meters have been used successfully in various human activity recognition applications [36, 37, 40, 41]. This includes uses in healthcare, behavior modelling, commerce, traffic condition monitoring, transportation identification, intelligent environments, fall detection, and to assess the wellness and fitness of athletes [37, 41–49]. Zhang et al. [50] and Akhavian and Behzadan [51] studied the use of smartphones for activity recognition and classification in the construction industry, showing the ability of smartphones to detect and define human-performed work activities. While sensor-based activity recognition has proven effective, model development and evaluation in the natural resource management field provides new opportunities to quantify and log both human and equipment-based work activities simultaneously within a common framework. Research on the development of consumer accessible smartphone and smartwatch-based activity recognition solutions for natural resource industries is lacking.

Previous activity recognition modelling using accelerometers, GNSS devices, and gyroscopes in natural resource industries has examined applications for motor-manual felling, motor-manual willow coppice operations, bandsaw mills, and carriages in cable logging operations [11, 52–55]. Pierzchala et al. [56] used a combination of smartphone derived IMU and GNSS data, video recording, and tethered onboard computers to perform automatic work phase recognition and subsequent quantification for a cable logging carriage with activity segmentation success of 78%. Keefe et al. [40] was the first successful use of stand-alone smartphone-based data collection for the development of human activity recognition models in forestry by assessing manual fallers in industrial forest operations. Using a smartphone secured to the belt of a timber faller, the activity recognition model characterized manual felling work elements and delay with accuracies between 65.9% and 99.6%. More recently, smartwatch sensors have been successfully used to develop activity recognition models for rigging crew workers setting and disconnecting log chokers on cable logging operations [57]. These new smart device-based activity recognition applications show promise for further integration into forest industries. There is a significant need for continued development of models for real-time production analysis and logistics. For new automated technologies to be used for time study research purposes, McDonald and Fulton [20] suggested several requirements need to be met: 1.) It must be unobtrusive, simple, and easy to implement; 2.) It must be applicable for a wide range of machinery and site conditions without need for major reconfiguring; 3.) It must be durable and robust; and 4.) It must produce data of comparable accuracies to that collected by a skilled field crew. Smartphones and smartwatches have been shown to provide a technological solution capable of meeting these evaluation metrics.

There is no currently published research on the development of stand-alone smartphone-based activity recognition models to predict work cycle elements and production rates of heavy equipment in forestry. Smartphones are useful for activity recognition model development due to their ubiquity, unobtrusiveness, ease of use, portability, high storage capacity, powerful computing, powerful embedded sensors, low energy consumption, extensive developer support and programmable capabilities [58–60]. Integrated production and logistic monitoring systems for collecting and processing data regarding machine and operator condition and performance are found in new forest equipment and exist for harvesters, forwarders, and other equipment [24, 25, 56]. However, in most instances in the United States, these are typically machine specific, proprietary hardware and software systems that are limited to a single machine, application, and manufacturer. Mobile activity recognition tools using smartphone-based IMUs present a standalone, mobile solution enabling integration, utilization, and customization for a wide range of mechanized equipment and work activities through a single device, similar to fitness tracking applications. Forest equipment movements entail unique, but significant, actions including lateral movements, swings, hydraulic boom manipulation, and hydraulic cutting or processing head operations. The distinct work elements and the cycles they comprise may enable the development of activity recognition-based applications based on equipment movements within their environments.

Accurately capturing, recording, and characterizing the elements of equipment is key to successful activity recognition development and opens the door to correlating equipment productivity analysis to environmental and site characteristics. The production and costs of mechanized felling, skidding, and processing equipment are directly impacted by forest stand metrics (tree size, stand density, undergrowth) and geophysical attributes (slope, rocks, debris, soils) [6, 8, 61]. Representing geographic and forest stand characteristics and their resulting impacts on equipment trafficability, tractability, and productivity is important for developing accurate, reliable, and robust activity recognition models for automating work activity characterization.

Remotely sensed data may support activity recognition model development by providing descriptive information related to site and stand conditions within the work environment. Light detection and ranging (lidar) is used extensively in forest, range, and fire management to quantify vertical forest structure, succession, and other forest attributes, as well as terrain morphology [62–70]. Use of lidar to conduct inventories and to characterize geophysical and ecological conditions across temporal and spatial scales can reduce inventory costs and improve the resolution of data for land management agencies and the forest industry [71]. In forest operations, lidar has been used to locate skid roads, log landings [64], detect disturbance related to harvest [72–74], quantify harvesting productivity [8], delineate harvest units and cable corridors [75, 76], and perform harvest system classification [77]. Additionally, Maltamo et al. (2019) assessed the accuracy of stand stem distributions derived using single-tree data from a harvester with an integrated positioning system and lidar metrics [78].

Area-based and individual tree-based approaches for deriving forest information from lidar data have been studied and compared extensively [79–83]. With improvements in lidar technologies and increased pulse densities, individual tree-based analysis has rapidly increased [84]. Pulse densities of 5–10 per square meter enable the efficient detection and segmentation of individual trees, and many current lidar acquisitions are easily within or exceed this pulse density [79]. Single tree detection and delineation is becoming a common tool for forestry applications including characterizing individual tree attribute distributions at the population level, modeling individual tree growth, classifying wildlife habitat, estimating carbon sequestration and other ecosystem services, modeling fire behavior, and precisely estimating biomass for inventory and operational applications [85–87]. Individual tree metrics including height, diameter at breast height (DBH), canopy base height, crown condition, crown area, biomass, stem volume, and specie can be derived from segmented tree data [64, 71, 79, 88]. The University of Idaho Experimental Forest has incorporated lidar-based single tree inventory into management operations for applied research, demonstration and teaching purposes since late 2020. However, to effectively use these high-resolution, remotely sensed data for operational forestry applications, it is also necessary to accurately position mobile heavy equipment during regular work conditions in the forest. Lidar-derived single tree products can have high geographic precision and accuracy, but positional data derived from GNSS-based devices can return high positional error. The inaccurate positioning of equipment or attachments in relation to remotely sensed products may lead to invalid assumptions regarding the relationship between these two data types.

The integration of smart devices monitoring equipment movements with lidar-derived single-tree information is critical to advance precision forest operations. Precision forestry, which commonly utilizes remotely sensed data sources and analysis, is the forest management technique emphasizing data-intensive and innovative practices, technologies, and processes to increase productivity, reduce costs, reduce negative site impacts resulting from management, and increase overall forest management success [89, 90]. These concepts are used in forest inventory and forest planning and have many opportunities for advancing industrial forest operations, as described above, while also allowing forest management planning and implementation at microsite levels [18]. Advances in precision forestry could provide greater insight into operational trends, processes, production, and machine interactions during logging operations for both production and safety analysis. In turn, this may lead to more sustainable, efficient, and safe forest operations. The expansion of smartphone and mobile-based internet of things (IoT) in forested environments through activity recognition will help evolve the definition, monitoring, and assessment of work in the woods.

This study focuses on the development and validation of a smartphone-based activity recognition modelling framework for forestry equipment. Our first objective is to develop equipment specific, smartphone-based activity recognition models for an excavator-based masticator using random forest machine learning algorithm. Our second objective is to evaluate the effects of sliding window sizes, window overlap proportion, and sampling frequency on activity recognition model performance. Lastly, we evaluated the accuracy of matching equipment body and attachment locations derived from consumer-grade GNSS-RF devices to lidar-derived individual tree locations during operational mastication work.

If successful, the development, validation, and future implementation of smartphone-based activity recognition models could assist land managers and operators by providing a ubiquitous system for continuous and unattended analysis of mastication treatments. Additionally, successful integration of lidar-derived single tree inventories and location-enabled activity recognition may facilitate the enhanced evaluation of forest and operational factors affecting treatment production and cost analytics in real-time. This will simultaneously provide data which can be used in planning and implementation of future forest operations and management. If successful, identifying masticated trees within a single-tree stem map could also foster accurate prediction of residual stand density, residual fuel characteristics, and other prescription features to inform and prioritize cost-effective long-term management planning scenarios.

## Materials and methods

### Study site

Three replicated stands on the University of Idaho Experimental Forest (UIEF) were sampled as part of a larger fuels management operation with treatment units located in three UIEF management units: Flat Creek; West Hatter; and East Hatter. The UIEF is located approximately 20 kilometers northeast of Moscow, Idaho in the Palouse Range. All sampled stands were approximately 27-year-old ponderosa pine (*Pinus ponderosa*) plantations established following a clearcut timber harvest and subsequent prescribed burn. The primary fuel treatment prescription was the reduction of stand density to a tree spacing of 5 x 5 meters using mastication. This pre-commercial treatment focused on reduction of stand density, treatment of western pine beetle damage, and hazardous fuels reduction for the residual stand.

### Mastication sampling and field procedures

A time and motion study was performed within the three stands to facilitate the observation of mastication work cycles with corresponding and simultaneously collected smartphone sensor data. Treatments were implemented with a tracked excavator (Kobelco ED150) running a hydraulic disk mastication head (Promac Brush Cutter). Due to hazardous conditions for observers during mastication and the density of the stands, observational sampling was supplemented with video recording captured by two Garmin Dash Cam 45 (Garmin®, Olathe, KA, USA) devices placed within the machine cab. These devices provided time-stamped video and audio recordings, enabling productive work cycle classification while increasing observer safety and reducing sampling errors associated with poor visibility, observer inattentiveness or element misclassification. GNSS-RF multi-transponder devices with 2.5 second sampling intervals were secured outside the cab of the equipment to record movements and treatment progression throughout sampling. Data recorded from these devices was shared in real-time via radio transmission to a handheld receiver.

**Fig 1:**
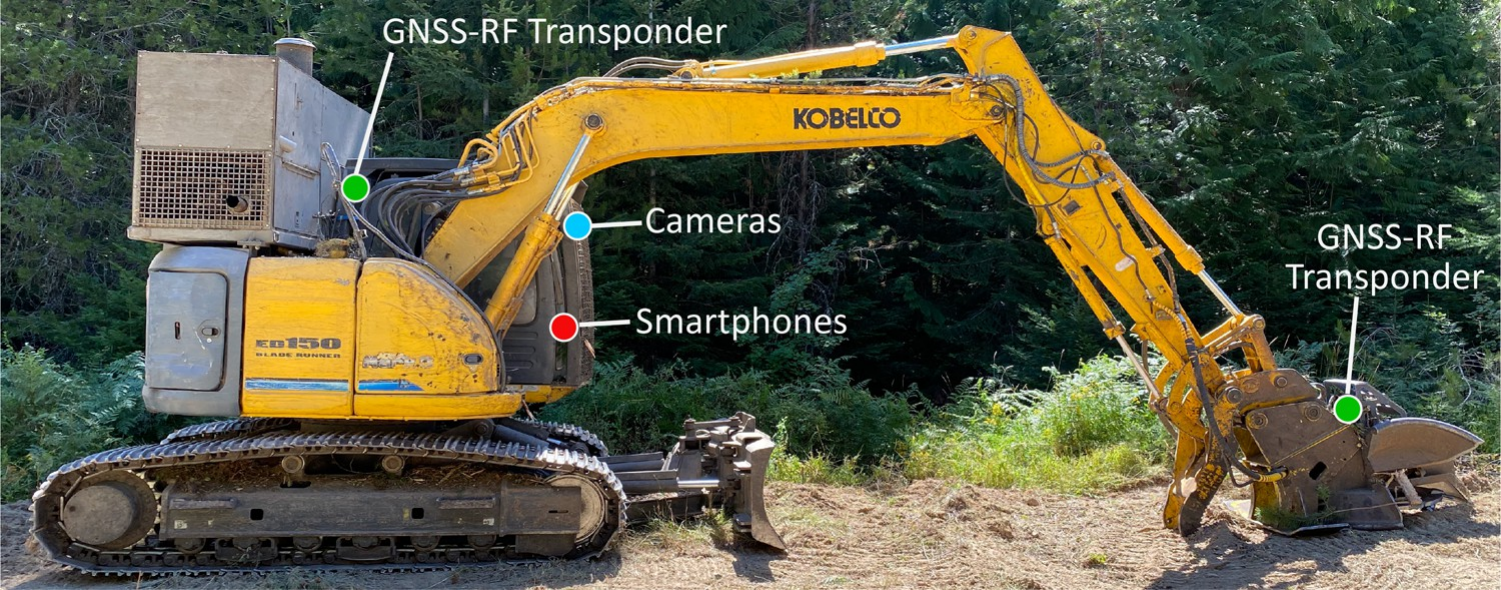
Smartphone, camera, and GNSS-RF transponder device locations on the masticator.

Google Pixel 2 smartphones (Google, Mountain View, CA, USA) collected the sensor data necessary for activity recognition modelling. These devices were placed within the machine cab oriented with the rear of the phones facing the front windshield. The AndroSensor application [91] was used to record the IMU and other smartphone sensor measurements. Within the AndroSensor application, a unique data sampling rate frequency was selected for each of the three phones used: 10, 20, and 50 hertz (Hz). The 10, 20, and 50 Hz sampling rates recorded information every 0.1, 0.05, and 0.02 seconds respectively. Device placement on the equipment used in field sampling is shown in Fig 1.

In human activity recognition, sampling frequencies vary greatly from 1 Hz [92] to 200 Hz [93, 94]. 50 Hz is often used to capture dynamic activities [58, 95], though 20 Hz has been shown to provide acceptable recognition accuracy while also preserving smartphone battery life [37, 45, 46, 96]. While decreasing sampling rate can increase the battery life of devices, reducing frequencies too drastically can limit the potential for achieving accurate activity recognition [92]. Collecting data at a high sampling rate (50 Hz) enables data to be downsampled to lower frequencies for additional analysis [60, 97, 98]. Therefore, the 50 Hz sensor data was downsampled to 20 Hz and 10 Hz to determine if downsampled data returned similar activity recognition classification results as natively sampled signals of the same frequencies.

Sensor data for acceleration, linear acceleration, orientation, and sound level was measured during field sampling using the Google Pixel 2. The IMU in the smartphone is a combination accelerometer and gyroscope that detects triaxial acceleration measurements in the x, y, and z directions in meters per second squared (m s^-2^), triaxial linear acceleration measurements in the x, y, and z directions in meters per second squared (m s^-2^), and triaxial orientation or tilt using the gyroscope in radians per second (rad s^-1^). Sound pressure levels were measured in decibels (dB) using an integrated sensor. Each recorded data point is assigned a date and time stamp indicating the year, month, and day and the associated hour, minute, second, and millisecond (Y-M-D h:m:s:SSS).

Productive work cycles and cycle elements were defined using the audio and video recordings obtained from the dash cameras used during sampling and were subsequently merged with smartphone-derived sensor data. Cycle elements for the masticator are described in Table 1 below.

**Table 1:** Cycle elements used for the mastication time and motion sampling.

Element	Description
***Move***	Starts when machine begins track or boom movement to successive masticating or clearing element and ends once head contacts material
***Masticate***	Starts when mulching head contacts standing tree and ends when tree bole is fully masticated.
***Clearing***	Starts when machine initiates mulching or moving downed trees, shrubs, etc. and ends when machine starts subsequent element
***Travel***	Prolonged machine walking within previously masticated area from one mastication location to the next
***Delay***	Any interruption to the productive cycle that falls outside previously described elements

### Activity recognition modelling and assessment

#### Smartphone-derived sensor data processing

Activity recognition modelling using random forests and other shallow learning algorithms primarily consist of four main phases: data collection; segmentation; feature extraction and selection; and classification [37, 59, 99]. Once data is collected, extracted from the smartphones, and work elements for the entirety of the data set are manually added to the data based on corresponding time stamps, data was imported into the R statistical programming environment where all remaining analysis and modelling occurred [100]. The segmentation phase of activity recognition entailed dividing the full data set into smaller time segments to simplify data retrieval. This process consisted of two steps: signal preprocessing and windowing [37, 59]. The Google Pixel 2 accelerometer and gyroscope report raw data in three axes (x, y, and z) enable the detection of orientation changes. To reduce the impact of orientation on activity recognition performance, the vector magnitudes of the triaxial sensors were calculated using Eq 1 [99, 101, 102]. This resulted in orientation-independent measures for acceleration, linear acceleration, and orientation/tilt.


$$a=\sqrt{{ax}^{2}+{ay}^{2}+{az}^{2}}$$ (1)

In activity recognition, sliding windows are often used to segment data into set time series where defined time domain features are calculated [37, 38, 43, 45, 46, 95, 103]. Studies have examined and compared model accuracies using multiple window sizes ranging from 1 to 13 seconds [40, 43, 45, 57, 92, 95, 103–110]. Activity recognition accuracy is greatly influenced by window size, with windows that are too short not covering the extent of the activity being classified and windows that are too long overlapping multiple unrelated activities [46]. Based on our study objectives and prior research, four different windows (1, 5, 7.5 and 10 seconds) were assessed to balance the ability of short windows to recognize simple activities and the ability of longer windows to capture less repetitive activities effectively [37, 40, 111]. Assigned window sizes are passed through the entire data set incrementally, with either overlapping or non-overlapping windows [112]. The strength of the impact that window size has on classification is dependent on the amount of overlap of subsequent windows [46]. While overlap enables the classification of activities in smaller time intervals than the window, it does require greater computational capacity than non-overlapping windows [104]. 50% window overlap is commonly used in activity recognition modelling [38, 43, 51, 95, 110, 111], though other window overlaps, including 90%, have been used successfully [40, 57, 105, 109]. Window overlaps of 50% and 90% were selected for this study.

Within each sliding window, time domain features were derived using mathematical and statistical techniques [113]. Nine time domain features were calculated: including the mean, maximum, minimum, kurtosis, variance, standard deviation, skewness, root mean square (RMS) and signal vector magnitude. These selected features were comparable with those used in Zhang et al. [50], Keefe et al. [40], Shoaib et al. [111], and Zimbelman & Keefe [57]. Mean is a versatile metric for various types of sensors for discriminating between rest and activity in multi-activity recognition and assists in preprocessing data by removing random spikes and noise [46, 113]. Similarly, RMS, signal vector magnitude and standard deviation are all effective statistics for activity classification and distinguishing between periods of rest and activity [46]. Skewness is used to measure “the degree of asymmetry of a probability density curve compared to the average” [50]. The purpose of sampling and data collection of various sensors is not to restore the raw signals of activities, but to detect different activities according to their statistical properties using the aforementioned time domain features [92].

#### Random forest

Following windowing, the data were separated into training and testing sets with a 70/30 split, respectively, to provide an unseen partition of data for final model assessment. Stratified random sampling was applied to each cycle element class to preserve element distribution ratios between testing and training data. Random forest (RF) was selected to develop machine learning algorithms for the productive cycle element classification. RF is an ensemble classifier that produces multiple decision trees using bagging and randomly selected subsets of training samples and variables to provide a majority vote from which a prediction is made [114–118]. Excellent classification results, speed of processing, and the ability to reduce bias, correlation, and overfitting compared to other classification and regression trees (CART) models make RF one of the most widely used machine learning methods available [118–121]. In activity recognition modelling, random forest has been widely tested with high levels of resulting classification success [40, 43, 47, 50, 57, 102, 103, 105, 108]. Performance was found to exceed 90% in instances when using multiple smartphone-based sensors and RF classifiers [36, 50].

**Fig 2:**
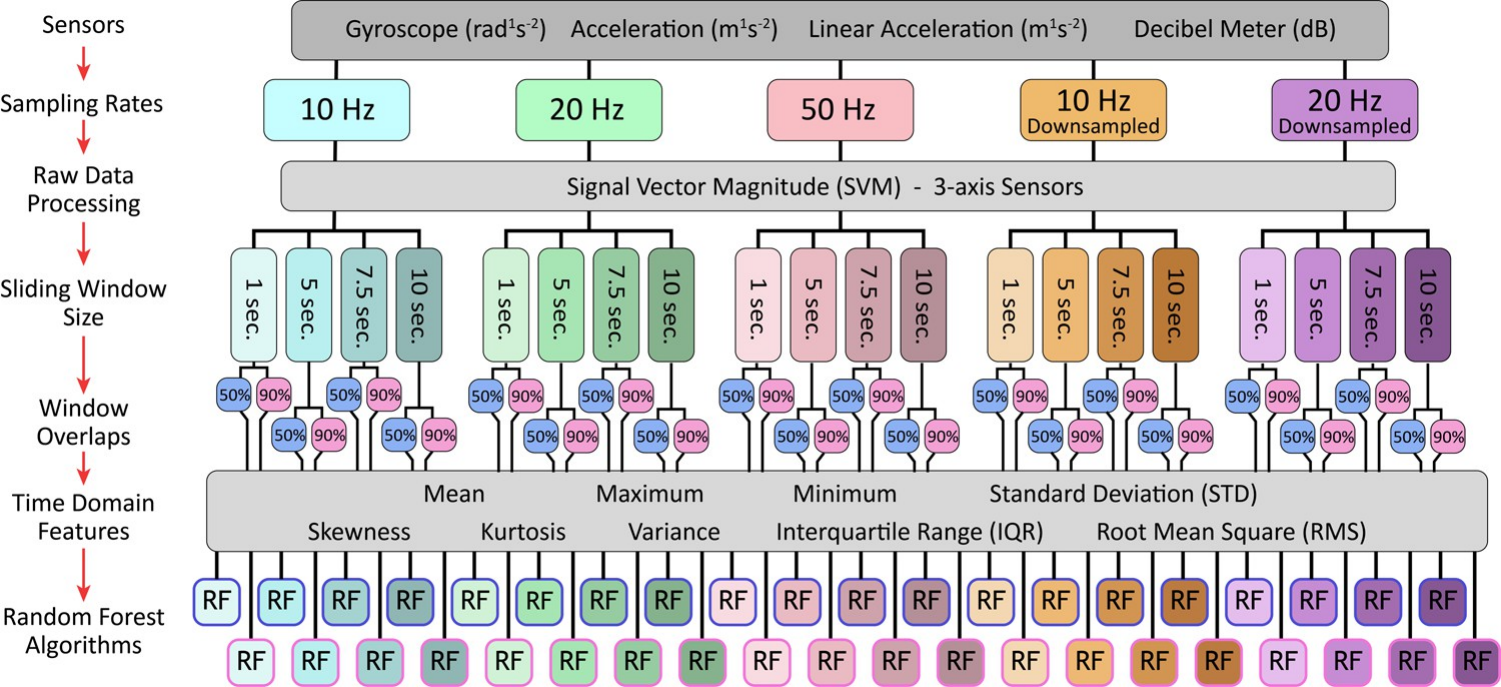
Activity recognition modelling data inputs and workflow.

Forty unique RF algorithms were developed to account for all combinations of sampling rate (10 Hz, 20 Hz, 50 Hz, 10 Hz downsampled, 20 Hz downsampled), window overlap (50% and 90%), and sliding window length (1 second, 5 seconds, 7.5 seconds, 10 seconds). The workflow for all data processing and model development is found in Fig 2. The R package *caret* (version 6.0–88) was used for all random forest training and development [122].

Hyperparameters, specifically *mtry* and *ntree*, are adjustable components of random forest which can increase the performance of algorithms when properly tuned. The *mtry* parameter determines the number of variables split at each node of the decision trees and *ntree* is the number of trees used in the forest [118]. Repeated k-fold cross validation with 10 folds and 3 repeats was used on training datasets to train and validate *mtry* and *ntree* values for the machine learning algorithms. *Mtry* values (1–25) and *ntree* values (1–550) were assessed during this process. The final selected value for *mtry* was chosen based on the maximum accuracy achieved. For *ntree*, it is understood that prediction accuracy increases at a decreasing rate as trees are added and larger values for *ntree* increase computational demands [114]. To balance processing time and model performance, *ntree* was selected by determining the point at which out of box error (OOB) and accuracy (%) of the model stabilized [123]. These hyperparameter values were then applied to the final training of the random forest algorithms which then predicted the test data.

The multiclass area under the curve (AUC) of the receiver characteristic operator (ROC) was used to evaluate the individual element characterization and overall activity recognition algorithm performance using the *multiclass*.*roc* function of the R *pROC* package (version 1.17.0.1) [124]. This measure of performance is commonly used for binary and multiclass classification algorithms [125–128] and performs well when working with imbalanced datasets [57, 126, 129–131]. The AUC for ROC represents the probability of a classifier ranking a randomly drawn positive before a randomly drawn negative [132]. In general, AUC provides an aggregate measure of classifier performance across all thresholds. This value falls between 0 and 1, with higher values indicating better performance [133]. AUCs between 0.5 and 0.7 are considered low accuracy; 0.7 and 0.9 are moderate accuracy; and accuracy is considered high when AUC exceeds 0.9 [125]. These values were converted to percentages. The AUC of all 40 trained random forest models were compared to determine which combination of window size, window overlap, and sampling frequency returned the highest AUC. The highest value indicated the best performing model overall.

Final model performance was also assessed using the *confusionMatrix* function in the *caret* package [122]. Five metrics quantifying the classification success of the activity recognition models per cycle element were provided by this function: sensitivity, specificity, precision, F1, and balanced accuracy. These accuracy metrics determined the impact of sliding window, window overlap, and sampling frequency on activity recognition performance. Sensitivity, also referred to as recall, is the true positive rate and represents the percentage of instances where an element was correctly classified [126]. This metric is calculated by dividing the number of true positives by the sum of the true positives and false negatives. Specificity is the true negative rate and is the percentage of negative instances correctly classified [126]. To calculate specificity, the number of true negatives is divided by the sum of the true negatives and false positives. Precision is used to capture the percentage of classified elements that represent an actual occurrence of that element and is calculated by dividing the number of true positives by the sum of the true positives and false positives [126]. In a basic sense, precision represents how sure one can be of their true positives, while sensitivity represents how sure one can be that no positive classifications are missed for a particular class. F1-score, or F-measure, combines the sensitivity and precision into a single performance metric and represents the harmonic mean of these two metrics [133–135]. This is the most common performance metric used in unbalanced classification problems [136–138]. The value of the F1-score ranges from 0 to 1, with values close to one indicating high classification performance [133], though they can easily be converted to a percentage scale and were in this study. Finally, the balanced accuracy was found for each model which combines the sensitivity and specificity metrics to find their average and works well with balanced and imbalanced data [133]. The equations for F1-score and balanced accuracy are found in Eqs 2 and 3 where F1 is the F1-score, and BA is the balanced accuracy.


$$F1=2*\frac{Precision*Sensitivty}{Precision+Sensitivity}$$ (2)

$$BA=\frac{1}{2}\;(Sensitivity+Specificity)$$ (3)

### Lidar point cloud processing procedures

Airborne laser scanning (ALS) is a commonly used lidar acquisition method for natural resource applications and derived products include terrain elevation, vegetation heights, stand volume, tree density, and tree species identification [68, 69]. The ALS data used in this study were collected using an Optech Galaxy Prime Lidar system mounted on a fixed-wing aircraft. This was part of a 9,363 km^2^ (3,615 mile^2^), multi-agency, state-wide, lidar acquisition performed by Atlantic LLC in October 2019. Nominal point spacing of 0.33 meters and an average nominal pulse density of 9.11 pulses m^-2^ was achieved. Individual tree detection and segmentation derived from the lidar point cloud was performed by Northwest Management Incorporated (NMI) using a proprietary software and processing workflow associated with NMI’s ForestView™ software [139]. This point-based lidar processing resulted in a full coverage ITD stem map for the four main University of Idaho Experimental Forest (UIEF) management units. In addition to individual stem coordinates, each predicted information associated with each tree included species, height (m), crown height (m), diameter (cm), crown diameter (m), and gross volume (MBF). It should be noted that the 9.11 pulses m^-2^ point cloud density used to develop the individual stem map using NMI’s ForestView™ software was an experimental evaluation using lower resolution pulse densities than are typically used to develop this commercial remote sensing product. Point densities of 16–20 pulses m^-2^ are typically used.

### Cycle element and individual stem location matching

Quantifying intensive equipment production that accounts for individual tree characteristics requires matching individual mastication events with single tree locations. The machine’s location for each mastication element was determined by aligning time series data for the GNSS-RF transponders and observed time and motion data. Once the equipment location for each mastication element was determined, it was then necessary to locate feasibly treated stems within the treatment zone of the masticator at that point in time. The equipment used had a maximum boom reach of approximately 9 meters. A circular buffer of 7 meters placed around the cab’s locations during mastication elements enabled the filtering of trees to include only those within the equipment’s reach. 7 meters was used because the mastication of standing trees does not enable full boom extension, thus limiting the overall reach of the machine.

**Fig 3:**
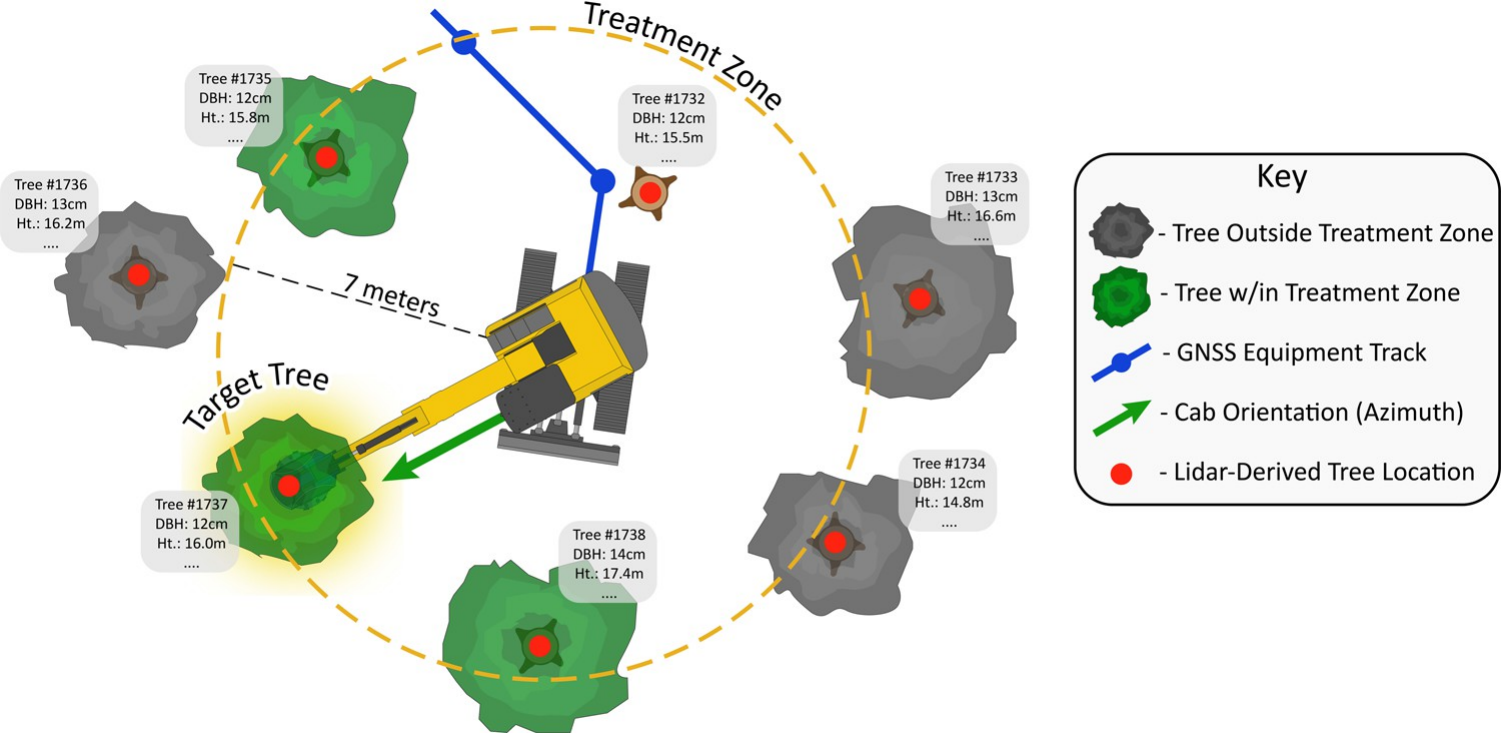
Identifying target trees. Process for identifying potential target trees for mastication using GNSS-RF-based equipment locations, equipment orientation, equipment to tree orientation, and individual tree locations.

Potential target trees were further filtered by accounting for the orientation of the machine cab within the stand in relation to the stems in the treatment zone. The cab and boom GNSS-RF devices enabled the derivation of the cab’s orientation as an azimuth. The azimuths between the equipment cab and each target tree within the associated buffer was then quantified. Cab-to-boom and cab-to-tree azimuths were compared, and it was assumed that cab-to-tree azimuths within +/- 10 degrees of cab-to-boom within the treatment zone indicated a feasibly masticated tree. All spatial analysis was performed using the sf packages in R [140]. Visual representation of the process used for identifying potentially treated trees using the stem map and GNSS-RF positions is shown in Fig 3.

## Results

### Work sampling and cycle analysis

Summarized results from the mastication work sampling data are found in Table 2. A total of 88,829 seconds (~24.7 hours) of mechanical fuel treatments were observed across the three treatment units, with units 1, 2, and 3 accounting for approximately 41%, 29%, and 30% of the sampling time, respectively. The equipment performed the *move* element most often during work sampling, accounting for 32.2% of the overall sampling time, which corresponds directly with the most individual occurrences of a single element, 1,348. *Delay* and *travel* occurred significantly fewer times than the other elements, accounted for the smallest percentages of total duration of sampled time, but also had the highest mean element duration, largest range of element durations, and the largest standard deviations in element durations. The *delay* element had the largest coefficient of variation (223.8%) indicating a large variation of values around the mean. This is, in part, a direct result of the large range of observed element durations with a total of 2,244 seconds (37.4 minutes) separating the longest and shortest elements. Total sampling duration for the *clear*, *masticate* and *move* elements were relatively equal and accounted for 26.0, 28.7 and 32.2% of the total work sampling time respectively.

**Table 2:** Work sampling summary statistics for mastication equipment by element for the full field sampling period.

Element	n	Total Duration (s)	Total Duration (%)	Mean (s)	Range (s)	SD (s)	CV (%)
Clear	932	23132	26.0	25.7	2.0–202.0	24.6	95.7
Delay	53	8417	9.5	211.5	14.0–2258.0	473.4	223.8
Masticate	921	25499	28.7	23.7	2.0–139.0	18.6	78.3
Move	1348	28626	32.2	20.2	2.0–207.0	17.7	87.7
Travel	33	3155	3.6	96.6	10.0–298.0	55.8	59.8
**Total**	**3287**	**88829**	**100.0**	**27.0**	**2.0–2258.0**	**56.1**	**207.7**

### Random forest and activity recognition

**Fig 4:**
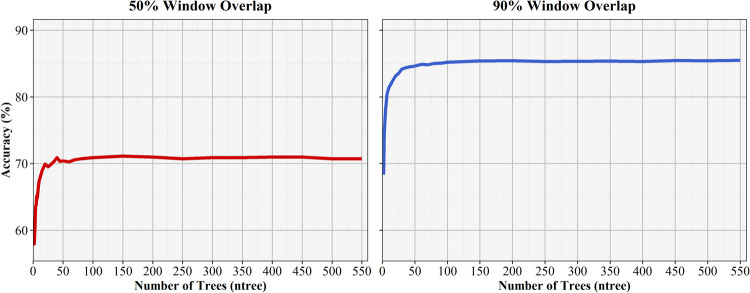
Repeated 10-fold cross validation ntree training results for the 50 Hz sampling rate and 10 second sliding window for both the 50 and 90% window overlaps.

Following the repeated 10-fold cross validation training and evaluation for the machine learning algorithms, 40 final random forest algorithms were evaluated using each combination of data sampling rate (Hz), sliding window size (seconds), and sliding window overlap (%). A *ntree* value of 150 was used for all random forests, with model training indicating a stabilization of error and accuracy at this number. This was found across all models, with training results for the 50% and 90% window overlap models for the 50 Hz sampling rate and 10 second sliding window algorithms shown in Fig 4. While larger *ntree* values provided slight increases in model performance, added benefits were outweighed by the significant increases in processing time. Increased processing time is a concern because of the delays caused in potential future real-time model applications. A *ntree* value of 150 provided an acceptable balance between model performance and processing time.

**Fig 5:**
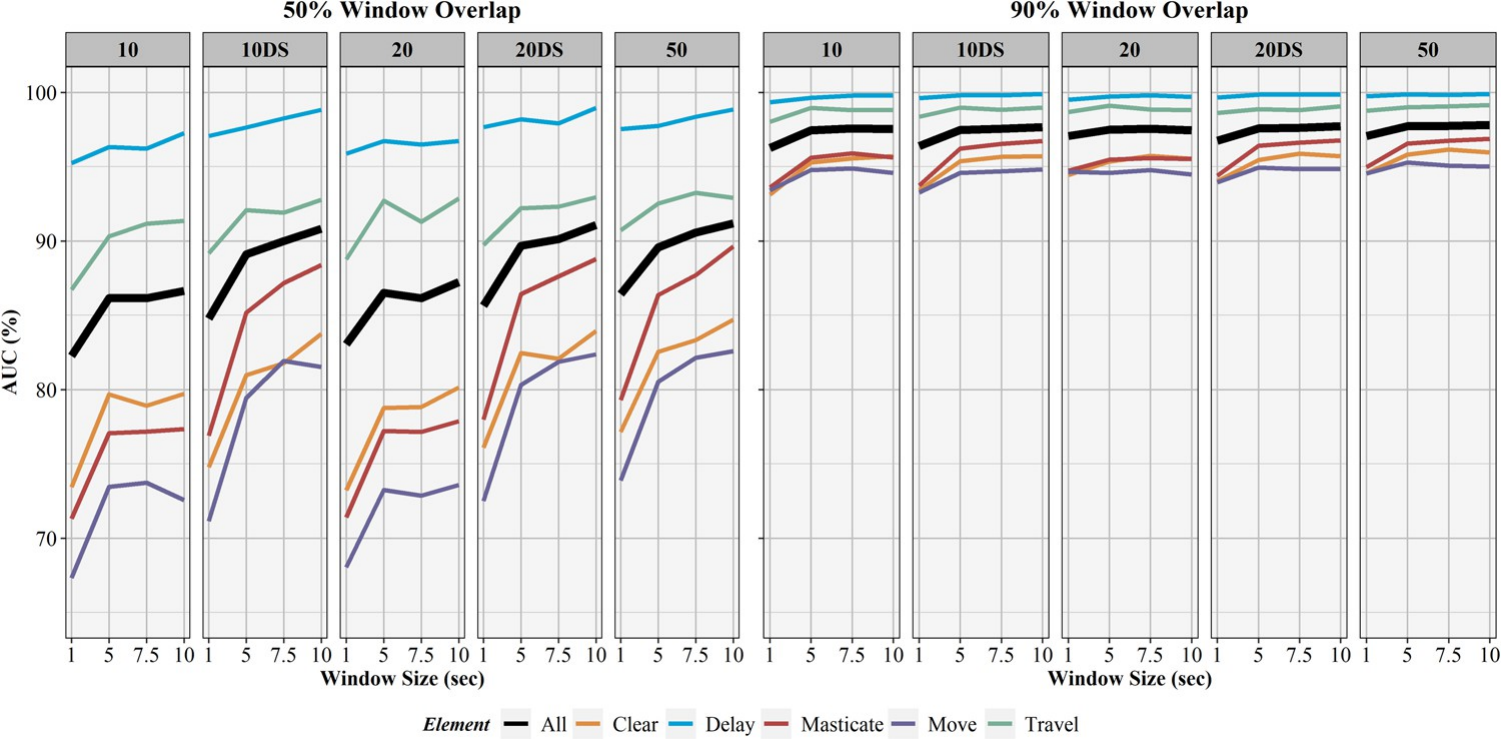
AUC results for activity recognition models. Comparison of activity recognition model area under the curve (AUC) percentages for 50% and 90% sliding window overlaps across the four sliding window lengths (1, 5, 7.5, 10 seconds) for each work element (clear, delay, masticate, move, travel) and for full models (All).

Overall activity recognition performance was quantified using the area under the curve (AUC) with results for all models (5 sampling rates, 4 window sizes, 2 window overlaps) displayed in Fig 5. In every instance, models with 90% window overlaps significantly outperformed models with identical sampling rates and window sizes but 50% window overlaps. Increasing sampling rate led to increases in AUC in most cases and similar trends were found when accounting for sliding window size. These trends were exaggerated in models with 50% sliding window overlaps. Final model selection was based on overall model AUC, with the best overall performance resulting from the 50 Hz sampling rate, 10 second sliding window, and 90% sliding window overlap combination with an AUC of 97.8%. AUC values for individual elements are found in Table 3.

**Table 3:** Activity recognition performance metrics for the best performing classification model (50 Hz sampling rate, 10 second window, 90% widow overlap).

Element	AUC (%)	Sensitivity (%)	Specificity (%)	Precision (%)	F1-score (%)	Balanced Accuracy (%)
Clear	96.0	82.2	93.5	81.5	81.9	87.9
Delay	99.9	95.6	99.8	98.3	96.9	97.7
Masticate	96.9	86.0	94.5	86.1	86.1	90.3
Move	95.0	84.2	90.8	81.1	82.6	87.5
Travel	99.2	74.9	99.9	98.1	84.9	87.4

**Fig 6:**
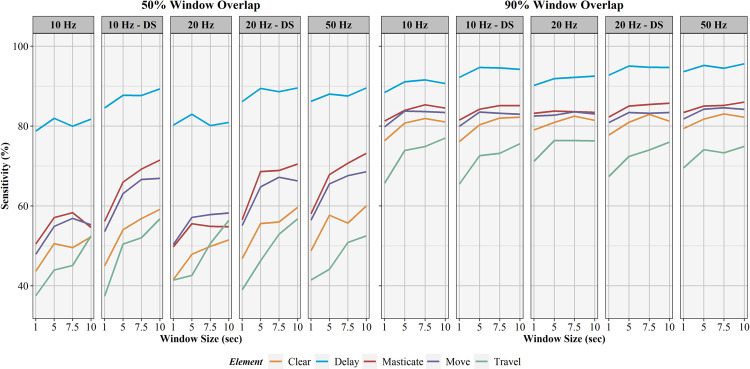
Sensitivity results for activity recognition models. Comparison of activity recognition model sensitivity percentages for 50% and 90% sliding window overlaps across the four sliding window lengths (1, 5, 7.5, 10 seconds) for each work element (clear, delay, masticate, move, travel).

**Fig 7:**
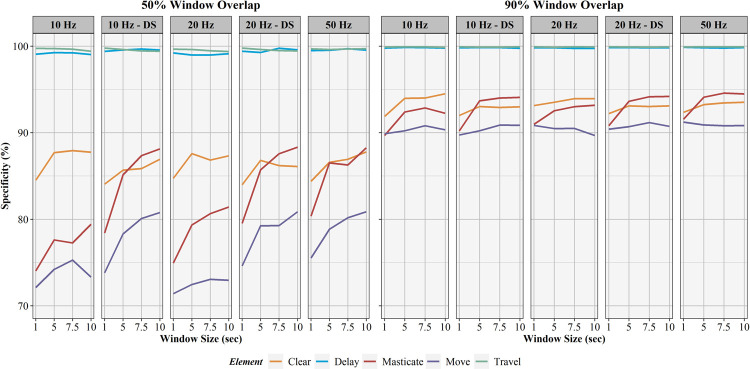
Specificity results for activity recognition models. Comparison of activity recognition model specificity percentages for 50% and 90% sliding window overlaps across the four sliding window lengths (1, 5, 7.5, 10 seconds) for each work element (clear, delay, masticate, move, travel).

**Fig 8:**
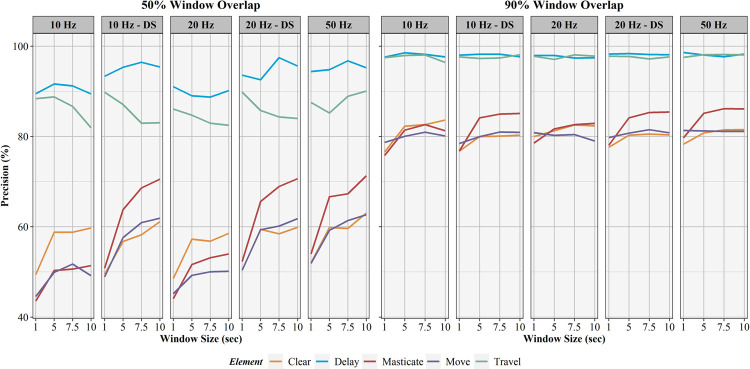
Precision results for activity recognition models. Comparison of activity recognition model precision percentages for 50% and 90% sliding window overlaps across the four sliding window lengths (1, 5, 7.5, 10 seconds) for each work element (clear, delay, masticate, move, travel).

**Fig 9:**
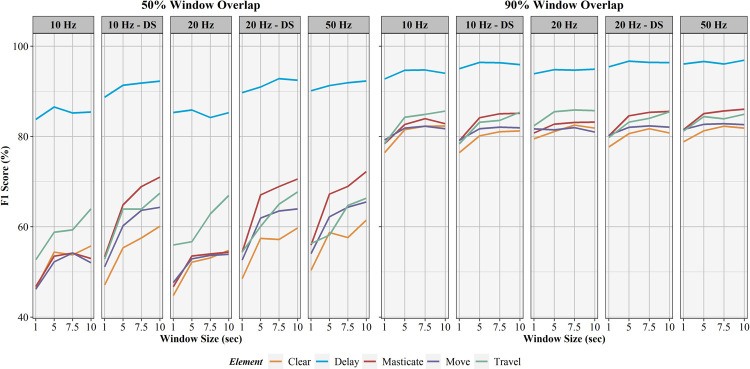
F1-score results for activity recognition models. Comparison of activity recognition model F1-score percentages for 50% and 90% sliding window overlaps across the four sliding window lengths (1, 5, 7.5, 10 seconds) for each work element (clear, delay, masticate, move, travel).

**Fig 10:**
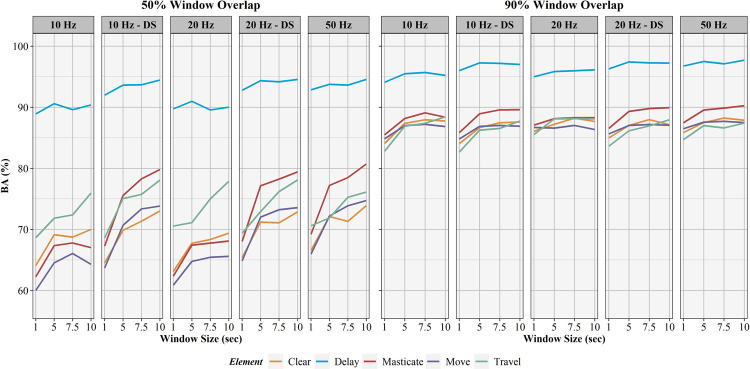
Balanced accuracy results for activity recognition models. Comparison of activity recognition model balanced accuracy percentages for 50% and 90% sliding window overlaps across the four sliding window lengths (1, 5, 7.5, 10 seconds) for each work element (clear, delay, masticate, move, travel).

The influence of sampling rate, window overlap, and window size on activity recognition sensitivity, specificity, precision, F1-score, and balanced accuracy are found in Figs 6–10, respectively. Like AUC, window overlap had the greatest impact on model performance for all metrics, with 90% models performing better than 50% overlap models. Increases in sampling rate provided marginal increases in model performance, with 10 and 20 Hz data downsampled from the 50 Hz data performing better than the natively sampled 10 and 20 Hz data for classification. Positive trends between increasing model performance and increasing sliding window size were also seen, with 10 second windows returning the highest performance metrics in most instances. Select work elements in some models returned higher performance metrics at 7.5 second windows than 10 second windows, but this was not consistent for all elements in these classification algorithms. Performance metrics for the best performing activity recognition model (50 Hz sampling rate, 10 second sliding window, 90% sliding window overlap) are found in Table 3.

The ranges of performance metrics used to assess the classification of the 5 work elements (*masticate*, *move*, *clear*, *delay*, *travel*) for the best model are as follows: AUC (95.0–99.9%); sensitivity (74.9–95.6%); specificity (90.8–99.9%); precision (81.1–98.3%); F1-score (81.9–96.9%); and balanced accuracy (87.4–97.7%). The *delay* element was correctly classified with the highest percentage of all elements and had sensitivity of 95.6%. *Travel* had the lowest sensitivity at 74.9%, but also achieved the highest specificity at 99.9%. The very high specificity means other work elements were rarely incorrectly classified as *travel*, though lower sensitivity shows actual *travel* events were incorrectly predicted as other elements relatively often, approximately 25% of the time. *Travel* element misclassification was most often associated with the *move* element, with 250 *travel* events incorrectly classified as *move* (Table 4). The *move* element had the lowest specificity at 90.8%, with 787 *clear*, 66 *delay*, 574 *masticate*, and 250 *travel* elements incorrectly classified as *move*. This shows the activity recognition model had the lowest ability to discern between false positives and true negatives with the *move* element.

**Table 4:** Confusion matrix. Confusion matrix for the best performing activity recognition model (50 Hz sampling rate, 10 second window, 90% window overlap) indicating true positives (TP), true negatives (TN), false positives (FP), and false negatives (FN).

		*Actual*				
		Clear	Delay	Masticate	Move	Travel
***Predicted***	**Clear**	5692	26	486	726	53
	**Delay**	1	2333	7	29	4
	**Masticate**	440	13	6570	588	18
	**Move**	787	66	574	7207	250
	**Travel**	2	3	2	12	970

**Fig 11:**
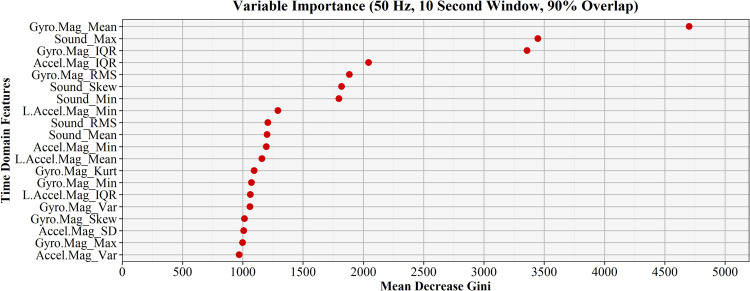
Time domain feature variable importance. Variable importance of the top 20 time domain features used during model development for the best performing activity recognition algorithm in terms of mean decrease in Gini.

The confusion matrix in Table 4 provides a detailed summary of activity recognition classification, with green shaded cells indicating correctly classified instances for each element. It is clear from the confusion matrix that the greatest misclassification occurred between the *move*, *masticate* and *clear* elements. The precision measures for all elements exceeded 80%, meaning over 80% of all elements predicted as a particular element were correctly classified. In all instances, the balanced accuracy exceeded 85%, showing the ability of the model to account for both positive and negative classification outcomes for elements as an average of specificity and sensitivity. Areas under the curve (AUC) exceeding 95% for all elements shows excellent overall classification accuracy and activity recognition performance for this model. Each individual event shown in the confusion matrix represents a single 10-second window used for activity classification. Models with smaller window sizes would contain larger numbers of instances within each cell because more windows were necessary to elapse the entire sampling period. The importance of the top 20 time domain features used in the final model are described by mean decrease in Gini and are found in Fig 11. Features from the gyroscope and sound derived features were consistently some of the most influential features in the models for all combinations of factors, consistent with the findings of Keefe et al. [40].

### Stem and cycle element matching

A total of 11,758 individual trees were detected within the treatment units, though work sampling did not encompass the entirety of the planned treatment area due to time constraints (Table 5). During the work sampling period, 1,140 individual trees fell within the 7m buffer of the equipment cab while masticating. Only 221 of these trees were within a range of azimuths from the cab to constitute a feasibly treated tree based on the GNSS-RF coordinates derived from the cab and boom transponders. In all, 921 mastication elements were observed during work sampling, though only 890 of these elements occurred with a lidar detected tree falling within the 7m cab buffer. 315 *masticate* elements could also be matched with an individual tree based on machine orientation at the time of mastication. More *masticate* elements (315) were matched with an individual tree than there were feasible unique trees (221) because *masticate* elements were matched with the same unique tree on multiple occasions. It can be assumed that once a tree is masticated, it can no longer be masticated again. Therefore, a maximum of 24% of all *masticate* elements (221) could be matched with a unique individual tree based on data derived from lidar-based individual tree locations and GNSS-RF-derived machine locations.

**Table 5:** Machine and individual stem location matching. This table displays the number of lidar-derived individual trees based on the ForestView™ software: within the overall treatment units; within the 7m treatment buffer of the machine cab during mastication elements; and within the cab buffer while corresponding with the machine orientation. The total number of mastication elements and the number of elements where an individual tree fell within the machine buffer and matched the machine orientation are also shown.

Lidar-derived Individual Trees			Mastication Elements		
Total	Buffer	Buffer + Azimuth	Total	Buffer	Buffer + Azimuth
11758	1140	221	921	890	315

## Discussion

Class imbalance has been shown to impact and bias classification results toward the majority classes [141]. The most common sampling methods to fix imbalanced data are over and under sampling [142]. Over sampling approaches resolve data imbalance by duplicating cases of the minority class but at the expense of computational intensity required for machine learning, and increased risk of model overfitting [142–144]. This leads to computationally demanding models. Under sampling entails the reduction of majority classes to meet minority class sample sizes but can lead to a loss of valuable data trends and information necessary for effective classification and was therefore not used in this study [137,143]. A third approach, synthetic minority oversampling technique (SMOTE), generates synthetic examples as opposed to data replication used in traditional oversampling methods, but are limited to binary classification models [143]. Ultimately, the data imbalance did not appear to have significant impacts on activity recognition model performance, even in the *delay* and *travel* minority classes, as seen in the performance metrics found in Table 3. Therefore, sampling procedures to address class imbalance in the data were forgone. This decision was further supported by Kamei et al. who found sampling techniques provided no performance benefit for classification tree models which encompasses random forest algorithms [145] and Dittman et al. [146] who concluded sampling procedures are not always a necessary step for classification because random forest classifiers’ robustness to imbalanced data.

While imbalance likely contributed to the slightly lower sensitivity of the *travel* element, this was not a concern, as the F1-score and balanced accuracy remained relatively high at 84.9 and 87.4%, respectively. The *travel* element is operationally similar in function to the *move* element, apart from element duration. These similarities in the *move* and *travel* elements may account for the comparatively poor sensitivity of the *travel* element. Here, data imbalance may have favored the majority class (*move*) over the minority class (*travel)* during classification. During classification, *travel* was misclassified as *move* more than any other element. The confusion matrix (Table 4) showed actual *travel* elements misclassified as *move* 250 times, which account for approximately 20% of the actual *travel* element occurrences and 77% of the total *travel* element misclassifications. In future work, the *travel* and *move* elements could be combined into a single element to simplify classification given their operational similarities. Primary productive cycle elements: *masticate*, *clear*, and *move*, returned sensitivities exceeding 80% and balanced accuracies exceeding 85% showing the strength of the model in classifying these work elements (Table 3).

Despite being a minority class, *delay* returned the highest performance metrics for AUC, sensitivity, precision, F1-score, and balanced accuracy among all work elements and the second highest specificity only behind the *travel* element. The machine was predominantly stationary during *delay*, simplifying the classification of this element and attributing to the comparatively high performance metrics. The limited impact of data imbalance identified in this study may vary with other datasets and should be examined further in future studies. The sensitivity (74.9–95.6%), specificity (90.8–99.9%), precision (81.1–98.3%), F1-score (81.9–96.9%), and balanced accuracy (87.4–97.7%) for element classification of the best performing activity recognition model were similar to those in other studies using random forest algorithms, though all other studies focused on human activity recognition [41, 47].

All time domain features were retained for the four sensors during modelling. To reduce the time and complexity of model processing for future iterations, the number of time domain features could be reduced by excluding less influential predictors. For example, the limited presence of linear acceleration in the top tier of time domain feature importance suggests this sensor data could potentially be excluded in future work without significant impacts to overall model performance. The five time domain features with the lowest importance were all derived from linear acceleration. The high importance ranking of the sound pressure meter derived time domain features is consistent with the findings of Keefe et al. [40]. Sound is a valuable measure for activity recognition for equipment as delay, lateral machine movements, clearing of light materials, and the mastication of standing stems all expectedly produce distinct levels of sound and should be included in future modelling efforts.

Window overlap percentage was the most influential factor associated with improved model performance for all combinations of sliding window length and sampling rate. The 50% window overlap was outperformed by the 90% window overlap by significant margins in all instances. This is consistent with findings from other studies [57, 109]. For example, the 50 Hz, 10-second sliding window model returned an overall model accuracy of 68.8% for the 50% window overlap and 84.8% for the 90% window overlap. One disadvantage of a greater window overlap is the added computational load of the model, with 90% overlap models taking longer to process than 50% overlap models. However, the increased performance metrics for activity recognition necessitates this computational cost to ensure effective and accurate models. Despite longer processing times, 90% window overlap can support real-time activity recognition by increasing the frequency at which predictions are made on the data and users receive activity characterizations [105].

Marginal improvements in performance metrics were obtained by increasing from 1, 5, and 7.5, to 10-second sliding windows for the 90% window overlap models. For example, the overall model AUC for 50 Hz, 90% window overlap models for the 1, 5, 7.5, and 10 second windows were 97.1, 97.7, 97.8, and 97.8, respectively (Fig 5). Window size impacted model performance more significantly for 50% overlap models than the 90% overlap models, which is supported by Khusainov et al. [46]. Overall AUCs for 50 Hz, 50% window overlap models for 1, 5, 7.5, and 10 second windows were 86.4, 89.6, 90.6, and 91.2, respectively. While 1, 5, 7.5, and 10 second windows can be used with similar effectiveness when using 90% window overlaps, the increased processing time required by the shorter window sizes should also be considered. Shorter sliding windows require high computational overhead because the recognition algorithm must classify more total windows than a larger window length would require over the same period of time [112]. The 10-second window provides strong model performance while minimizing this computational cost, which is an important consideration in real-time activity recognition. Increasing window length has also been shown to improve the performance of activity recognition models [40, 105, 111, 138]. This is contrary to findings of Zimbelman and Keefe who found better model performance with shorter window sizes when modeling work activities for rigging-crew workers on cable logging operations [57]. These findings may differ for other forest equipment where work element duration may favor shorter window lengths due to faster transitions between subsequent elements and the inability of longer window lengths to differentiate between short actions.

While limited, there were model performance improvements when moving from the 10 Hz and 20 Hz sampling rates to the 50 Hz rate. Highly active or “agitated” movements have been shown to be more accurately represented by higher sampling rates [37]. These improvements were more significant with the 50% window overlap models compared to the models with 90% overlap. A similar relationship was found with sliding window length. When accounting for only the models using a 90% window overlap, all models regardless of sampling rate returned strong values for AUC exceeding 90%, indicating strong classification success. The strongest classification models resulted from the 50 Hz rates. The increased performance of downsampled 10 and 20 Hz models over natively sampled 10 and 20 Hz models was likely a direct result of these data being a subsample of the 50 Hz data. The performance trends in relation to cycle element, window overlap, and window size mimicked those seen with the 50 Hz models, albeit with slight performance reductions.

Reduced battery consumption associated with sampling at lower frequencies [45, 96] was not found to be a necessary consideration in this study and computational resources needed for modelling were not impacted by sampling rate. During field sampling, all smartphones experienced similar battery draw when removed from auxiliary power, though longer periods of sampling and additional computational requirements associated with real-time activity recognition may show greater discrepancies. The use of equipment auxiliary electricity to power devices while sampling removes any concerns about sensor sampling frequency or other factors impacting battery life of devices during use. This is a distinguishing consideration when using smart devices for activity recognition on equipment versus human activity recognition where auxiliary power may be unavailable or cumbersome.

Integrating GNSS-RF derived machine positions and lidar-derived individual tree detection and segmentation to provide equipment positioning in addition to the smartphone activity recognition model was ultimately ineffective for our data. The ~25% success rate achieved matching mastication elements and individual stems is not operationally useful. The poor matching performance limits confidence in the stems which were matched, and further work should explore more effective and accurate means for this process. The exact reason for this poor performance is unclear and a combination of multiple factors may have led to the outcomes encountered. First, the accuracy of the individual tree detection (ITD) product used in this study has not been assessed. However, ITD accuracy of the ForestView™ algorithm was quantified in a separate study on the University of Idaho Experimental Forest using lidar data with an average pulse density of 20 pulses m^-2^ [139]. Recall of ITD ranged from 0.36 to 0.67 for varying canopy cover conditions and is comparable to other ITD algorithms [139]. It is assumed ITD recall for this study is likely less than that determined by Sparks and Smith [139] due to the lower pulse density of the lidar data used. It is possible that suppressed or intermediate trees were occluded or omitted during lidar-processing which would have reduced the number of detected stems, especially in areas of high stand density [147–150]. The mastication treatments primary focused on stand density reduction and stand improvement. Stems that were masticated were often smaller than those left in the residual stand. The use of terrestrial-based lidar may prove a more effective means of producing precise and accurate stems maps used for this application in future work. Mobile terrestrial laser scanning systems help reduce occlusion and omission and enable easier acquisition of multi-temporal lidar data sets [151, 152].

Increasing the precision and accuracy of the GNSS positioning of the equipment during the operation is another area for improvement in future work. Improving equipment positioning relative to individual stems detected from lidar would likely increase the accuracy of detecting removed stems. It is well established that forest canopies impact the accuracy of GNSS devices. However, the increased availability of consumer technologies to improve the GNSS accuracy of mobile devices provide new opportunities to enhance real-time positioning and logistics in forested environments. External smartphone antennas, raw GNSS data collection, availability of consumer-grade devices with differential correction and improved dual-frequency GNSS chipsets available for the newest generations of smartphones provide opportunities to greatly increase GNSS accuracy [153–159]. These technological advances may reduce the need for dedicated GNSS-RF devices and streamline activity recognition and positioning integration. This could in turn reduce variability associated with fusing sensor data from multiple devices.

## Conclusion

We have shown that models developed from the smartphone-based sensors placed in machine cabs can be used to characterize activities of mastication treatments to reduce fuels in forested stands with balanced accuracies for elements between 87.4% and 97.7%. As the first known smartphone-based activity recognition study for mechanical forestry equipment, it was necessary to examine multiple factors impacting recognition accuracy and set the foundation for future equipment activity recognition modeling research. By demonstrating the application of this technology for automated productivity analysis, we have advanced research supporting ubiquitous smartphone-based activity recognition solutions for forestry equipment. This work can be improved by further refining field sampling and modelling procedures and integrating new mobile technologies enabling the collection of sensor and accurate positional data from a single device. Additional field sampling with varying topographic and stand conditions, operators, and equipment types should also be completed to help quantify the extent to which these factors may impact activity recognition performance given the limited scope of conditions encountered in this study. Activity recognition of forest equipment using consumer available devices is an important advancement in the field of precision forestry and forest operations management. By identifying effective sampling and modelling processes, future work can focus on refining and optimizing data processing and model development and continue advancing toward real-time data support in smartphone applications. The current era of forest digitalization and smart and precision operational forestry necessitates land managers, operators, and researchers are equipped with the latest resources and technologies to successfully address complex forest management challenges. Accurate and efficient assessment of equipment productivity and work practices through activity recognition is valuable for advancing safer, more cost effective, and sustainable operational forestry supply chains.
